# SARS-CoV-2 Production in a Scalable High Cell Density Bioreactor

**DOI:** 10.3390/vaccines9070706

**Published:** 2021-06-29

**Authors:** Anna Offersgaard, Carlos Rene Duarte Hernandez, Anne Finne Pihl, Rui Costa, Nandini Prabhakar Venkatesan, Xiangliang Lin, Long Van Pham, Shan Feng, Ulrik Fahnøe, Troels Kasper Høyer Scheel, Santseharay Ramirez, Udo Reichl, Jens Bukh, Yvonne Genzel, Judith Margarete Gottwein

**Affiliations:** 1 Copenhagen Hepatitis C Program (CO-HEP), Department of Infectious Diseases, Copenhagen University Hospital–Hvidovre, 2650 Hvidovre, Denmark; anna.offersgaard@regionh.dk (A.O.); carlos.rene.duarte.hernandez@regionh.dk (C.R.D.H.); anne.finne.pihl@regionh.dk (A.F.P.); rcosta@sund.ku.dk (R.C.); pham@sund.ku.dk (L.V.P.); sfeng@sund.ku.dk (S.F.); ulrik@sund.ku.dk (U.F.); tscheel@sund.ku.dk (T.K.H.S.); santseharayra@sund.ku.dk (S.R.); jbukh@sund.ku.dk (J.B.); 2 Copenhagen Hepatitis C Program (CO-HEP), Department of Immunology and Microbiology, Faculty of Health and Medical Sciences, University of Copenhagen, 2200 Copenhagen, Denmark; 3 Esco Aster Pte Ltd., Singapore 486 777, Singapore; nandini.prabhakar@escoaster.com (N.P.V.); xl.lin@escoaster.com (X.L.); 4 Bioprocess Engineering, Max Planck Institute for Dynamics of Complex Technical Systems, 39106 Magdeburg, Germany; ureichl@mpi-magdeburg.mpg.de (U.R.); genzel@mpi-magdeburg.mpg.de (Y.G.)

**Keywords:** severe acute respiratory syndrome coronavirus 2, COVID-19, scalable bioreactor, CelCradle, packed-bed, whole virus vaccine, inactivated vaccine, animal component-free, Vero cells

## Abstract

The severe acute respiratory syndrome coronavirus 2 (SARS-CoV-2) pandemic has demonstrated the value of pursuing different vaccine strategies. Vaccines based on whole viruses, a widely used vaccine technology, depend on efficient virus production. This study aimed to establish SARS-CoV-2 production in the scalable packed-bed CelCradle^TM^ 500-AP bioreactor. CelCradle^TM^ 500-AP bottles with 0.5 L working volume and 5.5 g BioNOC™ II carriers were seeded with 1.5 × 10^8^ Vero (WHO) cells, approved for vaccine production, in animal component-free medium and infected at a multiplicity of infection of 0.006 at a total cell number of 2.2–2.5 × 10^9^ cells/bottle seven days post cell seeding. Among several tested conditions, two harvests per day and a virus production temperature of 33 °C resulted in the highest virus yield with a peak SARS-CoV-2 infectivity titer of 7.3 log_10_ 50% tissue culture infectious dose (TCID_50_)/mL at 72 h post-infection. Six harvests had titers of ≥6.5 log_10_ TCID_50_/mL, and a total of 10.5 log_10_ TCID_50_ were produced in ~5 L. While trypsin was reported to enhance virus spread in cell culture, addition of 0.5% recombinant trypsin after infection did not improve virus yields. Overall, we demonstrated successful animal component-free production of SARS-CoV-2 in well-characterized Vero (WHO) cells in a scalable packed-bed bioreactor.

## 1. Introduction

The coronavirus disease 2019 (COVID-19) pandemic caused by the novel beta-coronavirus severe acute respiratory syndrome coronavirus 2 (SARS-CoV-2) [1] has had a major impact worldwide with >173 million confirmed infections and >3.7 million deaths (as of 8 June 2021) [2].

Infection may be asymptomatic or cause a wide range of symptoms ranging from mild symptoms of the upper respiratory tract to severe pneumonia and multisystemic disease. Furthermore, following acute disease manifestations, individuals may suffer from post-acute sequelae of SARS-CoV-2 infection [3,4,5,6]. Vaccine development has been moving at an unprecedented speed [7]. Currently, there are 185 vaccine candidates in preclinical development and 102 candidates in clinical development (as of 8 June 2021) [8], utilizing a wide range of technologies [7,8,9]. Several vaccines are already licensed for emergency use. While some of these novel vaccines rely on previously less commonly used vaccine technologies, such as mRNA technology (e.g., BioNtech/Pfizer and Moderna vaccines) or viral vector technology (e.g., AstraZeneca and Johnson & Johnson vaccines), others rely on more traditional approaches such as inactivated whole virus technology (e.g., Sinopharm, Sinovac, and Bharat Biotech vaccines).

Historically, in most licensed viral vaccines, antigens are based on whole virus particles, which are either inactivated or attenuated [10,11,12,13]. Whole virus inactivated or attenuated vaccines have been successfully used to protect against various diseases, including the vaccines against measles, mumps, and rubella, chicken pox, influenza, hepatitis A, yellow fever, rabies and Japanese encephalitis. Whole virus vaccines represent known technologies and are considered safe for patients, with worldwide infrastructures supporting their production [10,12]. Notably, a clear advantage of whole virus vaccines over other types of vaccines is that they typically target an array of epitopes comprised by the viral particle [7]. Conversely, licensed SARS-CoV-2 mRNA vaccines and viral vectored vaccines utilize the SARS-CoV-2 Spike protein as vaccine antigen [7]. Finally, compared to mRNA vaccines, whole virus vaccines are easier to distribute and store as they only require refrigeration.

Efficient and scalable production of large quantities of viruses in cell lines suitable for vaccine production in animal component-free conditions is central to the manufacturing of inactivated and attenuated whole virus vaccines. Importantly, other applications also rely on availability of large amounts of viruses, such as viral ultrastructural studies, animal immunogenicity studies, vaccine challenge trials and diagnostic assays. At the outset of the present study, SARS-CoV-2 production in bioreactors had not yet been described. Thus, in this study, we established a process for COVID-19 vaccine manufacturing in Vero (WHO) cells cultivated in a single-use tide-motion bioreactor (CelCradle^TM^ 500-AP), one of the few scalable packed-bed bioreactors available [14] and an attractive option for large-scale cost-effective production.

## 2. Materials and Methods

### 2.1. Cell Culture

Vero (WHO) cells (ECACC no 88020401, equivalent to ATCC CCL81) (Nuvonis Technologies GmbH, Vienna, Austria), used for CelCradle^TM^ 500-AP experiments, were cultured in the serum-free medium OptiPRO SFM (Gibco, Grand Island, NY, USA) supplemented with 4 mM GlutaMAX (Gibco, Grand Island, NY, USA), 100 U/mL penicillin and 100 µg/mL streptomycin (Sigma, St. Louis, MO, USA) in T175 flasks (Nunc, Roskilde, Denmark) at 37 °C and 5% CO_2_. Cells were detached with TrypLE Express (1×) (Gibco, Grand Island, NY, USA) for 5–10 min. The enzymatic reaction was stopped by dilution with cell culture medium, pelleting of cells by centrifugation for 5 min at 200× *g*, and subsequent resuspension in cell culture medium. The cells were sub-cultured every 3–5 days until passage 20.

Vero E6 cells (gift from J.D., University of Lille), used for TCID_50_ assays, were cultured in Dulbecco’s Modified Eagle Medium (DMEM) (high glucose, GlutaMAX Supplement, pyruvate) (Gibco, Paisley, Scotland) supplemented with 10% fetal bovine serum (Sigma), 100 U/mL penicillin and 100 µg/mL streptomycin (Sigma) in T175 flasks at 37 °C and 5% CO_2_. Cells were detached with Trypsin-EDTA solution (0.05% *w/v*) (Sigma) for 5–10 min. The enzymatic reaction was stopped by dilution with serum-containing cell culture medium. The cells were sub-cultured every 2–4 days.

### 2.2. Virus Isolate

The virus seed stock used in this study was a passage 4 virus stock derived from the virus isolate SARS-CoV-2/human/Denmark/DK-AHH1/2020 [15]. Passage 1 and 2 were carried out in Vero E6 cells maintained in DMEM supplemented with 10% fetal bovine serum (Sigma), 100 U/mL penicillin and 100 µg/mL streptomycin (Sigma) [15]. Passage 3 and 4 were carried out in Vero (WHO) cells maintained in serum-free medium as described above. The passage 4 virus stock was characterized by infectivity and genome titers as well as deep sequencing as described below.

### 2.3. Cell Culture and Virus Production in the CelCradle^TM^ 500-AP

The CelCradle^TM^ 500-AP (Esco Aster Pte. Ltd., Singapore) is a single-use bioreactor with BioNOC™ II macrocarriers comprising the packed bed. This bioreactor applies the tide motion principle exposing the cells growing on BioNOC™ II macrocarriers to alternating cycles of nutrition and aeration. Sterile CelCradle^TM^ 500-AP bottles with working volumes of 500 mL used in this study were provided pre-packed with approximately 850 BioNOC™ II carriers. Initially, Vero (WHO) cells were expanded in T175 and T500 flasks (Nunc). Cell seeding was carried out as recommended [16]. Briefly, cells were harvested, centrifuged for 5 min at 200× *g* and resuspended in 50 mL cell culture medium. The CelCradle^TM^ 500-AP bottle was pre-incubated with 450 mL cell culture medium for 30–60 min at 37 °C and 5% CO_2_ prior to seeding of 1.4 × 10^8^–2.3 × 10^8^ cells (as specified for individual experiments). CelCradle^TM^ 500-AP stage settings during cell attachment were: Rising rate 2 mm/s, top holding time 20 s, down rate 2 mm/s and bottom holding time 0 s. Attachment was evaluated after 2.5–3.5 h using Equation (1):

(1) $$\mathrm{attachment}\;\mathrm{efficiency}=\left(1-\left(\frac{\mathrm{total}\;\mathrm{cells}\;\mathrm{in}\;\mathrm{suspension}}{\mathrm{total}\;\mathrm{cells}\;\mathrm{seeded}}\right)\right)\times 100.$$

CelCradle^TM^ 500-AP stage settings during cell culture and infection were rising rate 1 mm/s, top holding time 10 s, down rate 1 mm/s and bottom holding time 10 s.

The aim was to maintain the glucose concentration >8.3 mM, and the pH value in the range of 7.0–7.5. A combination of fed-batch and medium exchange was used to maintain glucose concentrations and pH levels in the cultures. Prior to infection, cell culture supernatant glucose concentration, lactate concentration, pH value, and in selected experiments glutamine (supplied as GlutaMAX, the measurements represent total glutamine, both free and available as GlutaMAX) and ammonia concentrations, were evaluated in the morning and evening with the Cedex Bio Analyzer (Roche, Mannheim, Germany) and the FiveGo F2 pH meter (Mettler Toledo, Columbus, OH, USA). Medium was exchanged up to twice per day (morning and evening) to maintain glucose concentration. When required, the pH value and glucose concentration were further adjusted by the addition of 45% glucose solution (Sigma) and 8% NaHCO_3_ solution (Sigma). Sampling and measurements were carried out either before or both before and after medium exchange and/or adjustment of glucose and pH, as specified. After infection, glucose concentrations were measured with the GlucCell System glucose meter (Esco Aster Pte Ltd.), and pH values were evaluated with pH-indicator strips (Merck, Darmstadt, Germany). Cell culture glucose consumption between two time points was calculated as the difference between the total amount of glucose available in the cell culture supernatant upon each measurement. Glucose consumption recorded for one day was summed to calculate glucose consumption rate (GCR). The cell-specific GCR (csGCR) was calculated by dividing the total glucose consumption per day by the total cell number per day.

Cells were counted every day until the time of infection. To this end, five carriers were sampled from the 500-AP culture bottle and incubated 20–35 min with Trypsin-EDTA solution (Sigma) for cell detachment. Detached cells were collected in DMEM containing 20% fetal bovine serum. Cells were counted with a Scepter™ 2.0 Cell Counter (Merck) or with a hemocytometer with Trypan Blue (Gibco, Grand Island, NY, USA). The number of cells detached per carrier was multiplied with the estimated number of carriers in the 500-AP culture bottle (assuming 850 carriers upon start of culture, according to the product manual, and subtracting 5–6 carriers per day removed to obtain cell counts) to obtain total cell number in the CelCradle^TM^ 500-AP culture.

Infection was carried out when the total cell number exceeded 2 × 10^9^ cells with a prior medium exchange. The virus inoculum (passage 4 virus seed stock diluted in a total volume of 50 mL cell culture medium) was added to the culture at a multiplicity of infection (MOI) of 0.006. Then, cultures were maintained at 37 °C or 33 °C and harvests (approximately 90% medium exchange) were carried out 1 or 2 times per day as specified for individual experiments. In one specified experiment, the culture medium contained 0.5% (*v/v*) TrypLE Select 10× (Gibco, Grand Island, NY, USA) from the time of infection. Virus infectivity titers in the CelCradle^TM^ 500-AP harvests were determined using 50% tissue culture infectious dose (TCID_50_) assays as described below.

### 2.4. Virus Titration

SARS-CoV-2 infectivity titers were determined in a 96-well-based TCID_50_ assay evaluated by immunostaining of SARS-CoV-2 Spike protein subunit 1 (S1) [15,17,18]. Briefly, a 10-fold virus dilution series was prepared in DMEM and 100 µL per well were added in four replicates to Vero E6 cells seeded the previous day in a flat-bottom 96-well plate (Nunc) at 1 × 10^4^ cells/well. Cells were fixed 48 h post-infection (hpi) (+/−1 h) by 20 min incubation in cold methanol and subsequently washed in phosphate-buffered saline (PBS) with 0.1% tween-20 (Sigma). Endogenous peroxidases were inactivated by incubation with 3% H_2_O_2_ for 10 min. Blocking was done by incubation with PBS containing 1% (*w/v*) bovine serum albumin (Roche) and 0.2% (*w/v*) skimmed milk (Easis, Aarhus, Denmark) for 30 min. Cells were stained with primary antibody against S1 (Sino Biologicals, Beijing, China, #40150-D004) diluted 1:5000 for 2 h at room temperature or overnight at 4 °C. Subsequently, cells were stained with secondary antibody F(ab′)2-Goat anti-Human IgG Fc Cross-Adsorbed Secondary Antibody, HRP (Invitrogen, Waltham, MA, USA) or pre-adsorbed Goat F(ab′)2 Anti-Human IgG–Fc (HRP) (Abcam, Cambridge, UK) diluted 1:2000 for 1 h at room temperature and visualized with Bright-DAB solution kit (Immunologic, Duiven, The Netherlands). The 96-well plates were imaged with an Immunospot series 5 UV analyzer (CTL Europe GmbH, Bonn, Germany) [19]. The TCID_50_ was calculated with the Reed-Muench method [20].

The number of TCID_50_ units obtained from each harvest was calculated from the geometric mean of three replicate harvest infectivity titrations and the harvest volume. The accumulated TCID_50_ value obtained from each experiment was determined as the sum of TCID_50_ units in each harvest. The values given are rounded to one decimal. The cell-specific virus yield (CSVY) was calculated dividing the accumulated TCID_50_ units by the total cell number in the CelCradle^TM^ 500-AP recorded on the day of infection. Non-rounded accumulated TCID_50_ values were used for these calculations. Similarly, the fold-differences of accumulated TCID_50_ values obtained from different experiments were calculated based on non-rounded values.

### 2.5. RNA Titration

Pre-diluted virus-containing cell culture supernatant was mixed 1:3 with Trizol LS (Life Technologies, Carlsbad, CA, USA) and RNA was extracted with chloroform (Sigma) in 5PRIME Phase Lock Gel Heavy tubes (Quantabio, Beverly, MA, USA). RNA was purified with the RNA Clean and Concentrator-5 kit (Zymo Research, Irvine, CA, USA) according to the manufacturer’s instructions and eluted in nuclease-free water (Ambion, Austin, TX, USA). Primers and probes have been described elsewhere [21] and the reaction composition was adapted to TaqMan Fast Virus 1-Step Master Mix (Thermo Fisher). Briefly, 400 nM E_Sarbeco_F (5′-ACAGGTACGTTAATAGTTAATAGCGT-3′), 400 nM E_Sarbeco_R (5′-ATATTGCAGCAGTACGCACACA-3′) and 200 nM E_Sarbeco_P (FAM-5′-ACACTAGCCATCCTTACTGCGCTTCG-3′-BHQ1) and 2.5 µL purified RNA was used. In each assay, a negative control and RNA standards ranging from 10^1^ to 10^5^ RNA copies/µL (Twist Bioscience, South San Fransisco, CA, USA) were included. Two technical replicates of all experimental samples and controls were analyzed. Thermal cycling was performed at 55 °C for 10 min for reverse transcription, followed by 95 °C for 3 min and then 45 cycles of 95 °C for 15 s and 58 °C for 30 s using the LightCycler 96 System (Roche). RNA titers (copies/mL) were calculated by interpolation of cycle threshold (Ct) values in the standard curve generated from the standard panel, using the LightCycler 96 software version 1.1.0.1320 (Roche).

### 2.6. S1 ELISA

The amount of S1 obtained from harvests of experiment 7 was determined. Virus harvests collected at 36–122 hpi were pooled and inactivated chemically using β-propiolactone (details on inactivation will be reported elsewhere). The sample pools were evaluated by ELISA. 96-well Maxisorp plates (Nunc) were coated with 2.5 µg/mL capture antibody (Sino Biologicals #40150-D003) diluted in PBS. Plates were shaken at 500 rpm for 1 min and incubated overnight at 4 °C and washed three times with PBS 0.1% tween-20. Blocking was done by incubation with PBS containing 2% (*w/v*) bovine serum albumin (Roche) for 2 h at room temperature. The plates were washed three times with PBS 0.1% tween-20 followed by the addition of serially diluted recombinant S1 standards (Sino Biologicals #40591-V08H) or samples in duplicates. Plates were incubated at room temperature for 1.5 h with shaking at 500 rpm and washed five times with PBS 0.1% tween-20. Detection antibody (Sino Biologicals #40150-D001-H) diluted 1:5000 in PBS containing 2% (*w/v*) bovine serum albumin and 0.1% tween-20 was added, and plates were incubated at room temperature for 1.5 h with shaking at 500 rpm followed by washing five times with PBS 0.1% tween-20. Plates were incubated 5–10 min with TMB substrate and stop solution was added. Absorbance was measured at 450 nm (OD450) with a plate reader (BIO-TEK Instruments Inc., Winooski, VT, USA). Absorbance of the negative controls was subtracted from all measurements prior to analysis. The standard curve was generated using a four-parameter logistic regression in GraphPad Prism 9, and S1 sample concentrations were calculated based on the standard curve.

### 2.7. Deep Sequencing

Viral RNA was extracted as described above for RNA titration. Reverse transcriptase polymerase chain reaction (RT-PCR) and PCR conditions and primers are described elsewhere [15]. Amplicons were purified with the DNA clean and concentrator-25 kit (Zymo Research) according to the manufacturer’s instructions. The NEBNext Ultra II DNA library Prep kit (New England Biolabs, Ipswich, MA, USA) was used for library preparation and sequence analysis was carried out as described elsewhere [15,22,23]. Mutations prevalent in <5% of the virus population are not reported, and mutations with <50% prevalence are considered minor.

## 3. Results

### 3.1. Similar Cell Expansion Obtained Comparing Two Different Cell Numbers for Seeding of the CelCradle^TM^ 500-AP (Experiment 1, 2 and 3)

For all CelCradle^TM^ 500-AP experiments described in this study, following the seeding of the specified number of cells, the cell attachment efficiency was ≥98% after 2.5–3.5 h (Equation (1)). For all CelCradle^TM^ 500-AP cultures, either not subjected to infection (experiments 1–3) or prior to infection with SARS-CoV-2 (experiments 4–8), the medium was exchanged every day from the first or second day post cell seeding (dpcs) and twice per day from 4 dpcs. Additionally, NaHCO_3_ solution and glucose were supplied to the culture medium as required. In non-infected cultures, the pH value and concentrations of glucose, lactate, and in selected experiments glutamine and ammonia were measured and reported twice per day. Each time, the cell culture supernatant was sampled before and after medium exchange or adjustment of pH or glucose for calculation of consumption of nutrients and production of waste products between two time points.

**Figure 1:**
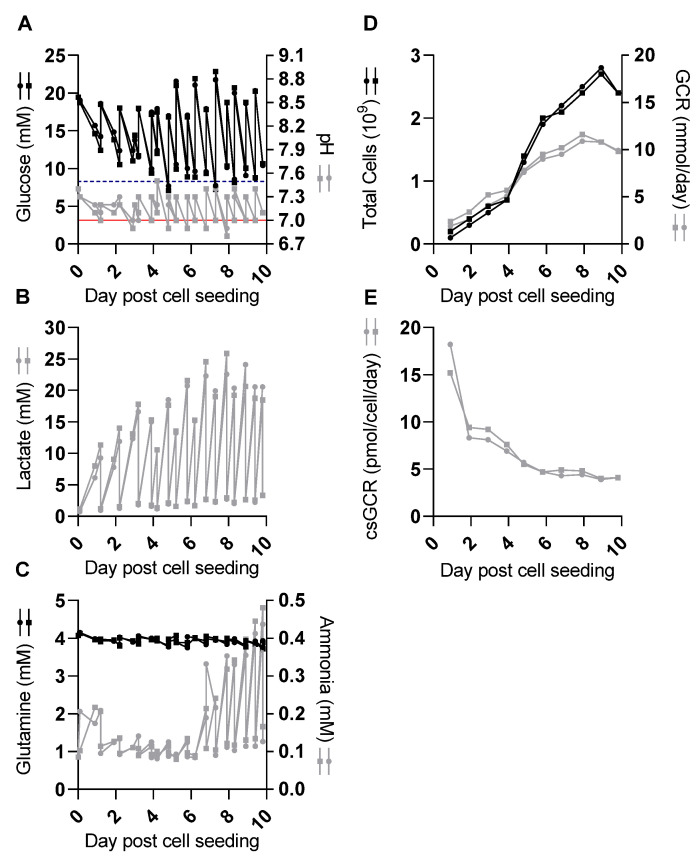
Cell expansion was similar using two different cell numbers for seeding of CelCradle^TM^ 500-AP bottles (experiment (exp.) 1 and 2). Two 500-AP culture bottles were seeded with a low (Seed_Low_, 1.4 × 10^8^ cells, circles) and a high (Seed_High_, 2.3 × 10^8^ cells, squares) seed cell number. Cell culture supernatant glucose concentrations and pH values were evaluated in the morning and evening and were adjusted by medium exchange and/or addition of glucose and NaHCO_3_ solution. pH values and concentrations of glucose, lactate, glutamine (supplied as GlutaMAX), and ammonia were measured before and after adjustment at each time point. Cell culture medium was exchanged every day, and twice per day from 4 dpcs. Carriers were sampled every day to determine total cell numbers. (**A**) Cell culture supernatant glucose concentrations (black symbols) and pH values (grey symbols); the dashed blue horizontal line represents a glucose concentration of 8.3 mM, the red horizontal line represents a pH value of 7, indicating the desired lower limits for these parameters. (**B**) Cell culture supernatant lactate concentrations. (**C**) Cell culture supernatant concentrations of glutamine (black symbols) and ammonia (grey symbols). (**D**) Total cell numbers per CelCradle^TM^ 500-AP calculated from a sample of five carriers (black symbols) and glucose consumption rate (GCR) (grey symbols). (**E**) Cell-specific GCR (csGCR).

Vero cells were first cultivated in the CelCradle^TM^ 500-AP bioreactor system without SARS-CoV-2 infection to evaluate cell growth and peak total cell numbers. Two seed cell numbers (1.4 × 10^8^, Seed_Low_, experiment 1 and 2.3 × 10^8^, Seed_High_, experiment 2) were initially compared (Figure 1). In the Seed_Low_ culture, the glucose concentration was in the range of 7.7–22 mM throughout the time of culture and was below the lower target of 8.3 mM before medium exchange at 5 and 7 dpcs. pH values were in the range of 6.9–7.5 throughout the time of culture, thus pH values were below the lower target of 7 before medium exchange at 5–8 dpcs (Figure 1A, Table 1). Daily peak concentrations of lactate increased to 24 mM at 9 dpcs (Figure 1B, Table 1). Glutamine concentrations (supplied as GlutaMAX) were in the range of 3.5–4.2 mM and daily consumption was relatively low peaking at 0.14 mmol at 9 dpcs. Ammonia concentrations up to 0.4 mM were observed at 9 and 10 dpcs (Figure 1C, Table 1). In the Seed_High_ culture, similar cell culture supernatant parameters were recorded (Figure 1, Table 1).

Comparing experiment 1 and experiment 2 with different seed cell numbers, there was only a minor difference in total cell numbers throughout the time of culture (Figure 1D, Table 1). Total cell numbers peaked at 9 dpcs with 2.8 × 10^9^ cells (3.5 × 10^6^ cells/carrier) and 2.7 × 10^9^ cells (3.4 × 10^6^ cells/carrier) in the Seed_Low_ and Seed_High_ culture, respectively. Similarly, only a minor difference in GCR was seen, with the Seed_High_ culture having a slightly higher GCR throughout most of the culture period. The GCR peaked at 9 dpcs with 11 mmol/day in the Seed_Low_ culture and at 8 dpcs with 12 mmol/day in the Seed_High_ culture (Figure 1D, Table 1). The csGCR was highest on the early days post cell seeding and then seemed to plateau at 4–5 pmol/cell/day from 6 dpcs onwards (Figure 1E). A third culture (experiment 3) (Table 1) initiated with a low seed cell number (1.5 × 10^8^ cells) demonstrated similar cell growth and culture parameters compared to the first experiment with peak total cell numbers of 2.7 × 10^9^ (3.4 × 10^6^ cells/carrier) at 9 dpcs. Thus, we concluded that there were no differences for the two seed cell numbers tested regarding peak total cell numbers and the number of days required for cell expansion. Since glutamine did not seem to be a limiting factor, glutamine and ammonia concentrations were not monitored in subsequent experiments.

### 3.2. SARS-CoV-2 Production with One Harvest Per Day (Experiment 4)

**Figure 2:**
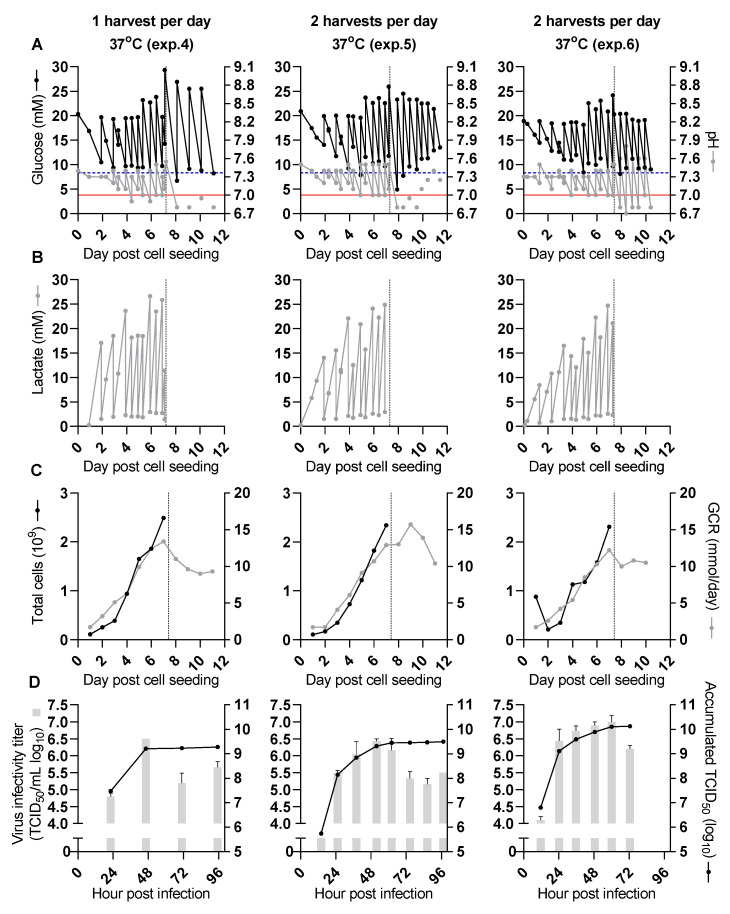
Higher virus yield obtained in SARS-CoV-2 production at 37 °C with two as compared to one harvest per day (experiments 4, 5 and 6). All culture bottles were seeded with 1.5 × 10^8^ cells per bottle. Before the time of infection (dashed vertical line) pH values and concentrations of glucose and lactate were measured before and after adjustment at each time point. After infection, glucose concentrations and pH values were measured before harvest (experiments 4 and 5, left and middle panel) or before and after each harvest (experiment 6, right panel). Harvest frequency is indicated in the figure. The three experiments were done independently. (**A**) Cell culture supernatant glucose concentrations (black dots) and pH values (grey dots). The dashed blue horizontal line represents a glucose concentration of 8.3 mM, the red horizontal line represents a pH value of 7, indicating the desired lower limits for these parameters. (**B**) Cell culture supernatant lactate concentrations. (**C**) Total cell numbers per CelCradle^TM^ 500-AP calculated from a sample of five carriers (black dots) and GCR (grey dots). (**D**) Infectivity titers of individual harvests (grey columns, showing the geometric mean of triplicate titrations and error bars indicating standard error of the mean) and SARS-CoV-2 yield expressed as accumulated 50% tissue culture infectious dose (TCID_50_) units (black dots, sum of TCID_50_ units obtained from each harvest of an experiment).

In all the following experiments, CelCradle^TM^ 500-AP cultures for SARS-CoV-2 infection and production were seeded with 1.5 × 10^8^ cells. The monitored pH values, concentrations of glucose and lactate, as well as total cell numbers and GCR, showed similar trends as to what was observed in initial experiments until the time of infection (Figure 2A–C, Table 1). Infection was carried out at the MOI of 0.006 at 7 dpcs when the total cell numbers exceeded 2 × 10^9^ (2.5 × 10^6^ cells/carrier) (Figure 2C, Table 1). This MOI was selected based on observations in another study [24], as well as on a pilot experiment in T25 flasks. In cultures infected with this MOI, virus-induced cell death was generally not observed one day post-infection (dpi); therefore, infections of the CelCradle^TM^ 500-AP cultures were carried out before peak total cell numbers were expected based on the initial CelCradle^TM^ 500-AP experiments.

The virus seed stock used to infect the CelCradle^TM^ 500-AP cultures in this study was a passage 4 polyclonal stock derived from a SARS-CoV-2 isolate from a Danish patient [15]. Compared to the original patient isolate, viruses in the passage 4 seed stock had acquired four minor (prevalence between 5 and 50%) substitutions all localized in the S protein. These substitutions were H66R with an estimated prevalence of 47% in the viral population, D215G with a prevalence of 12%, H245R with a prevalence of 17%, and S247R with a prevalence of 5% (Table 2). The virus seed stock was produced in a T175 flask and had an infectivity titer of 6.4 log_10_ TCID_50_/mL and an RNA titer of 9.7 log_10_ copies/mL.

The first CelCradle^TM^ 500-AP culture infected with SARS-CoV-2 for virus production was maintained at 37 °C during the virus production phase. Virus harvest and medium exchange were carried out once per day on 1–4 dpi. After infection, pH values and glucose concentrations were only measured before harvest and medium exchange, and lactate concentrations were not evaluated (Figure 2B). Additional NaHCO_3_ and glucose were added with each harvest and medium exchange. This was insufficient to maintain pH values or glucose concentrations above the lower target levels (Figure 2A, Table 1). It was expected that the addition of even larger volumes of NaHCO_3_ would leave pH values inappropriately high in the first hours after harvest and medium exchange.

The peak infectivity titer of 6.5 log_10_ TCID_50_/mL was observed for harvest 2 at 46 hpi (Figure 2D). The accumulated TCID_50_ from all harvests (2 L) was 9.3 log_10_ TCID_50_, resulting in a CSVY of 0.8 TCID_50_/cell (Table 3).

### 3.3. Improved Virus Yields by Two Harvests per Day (Experiments 5 and 6)

To evaluate whether increasing the harvest frequency to two harvests per day would increase virus yield from the CelCradle^TM^ 500-AP culture, virus harvest and medium exchange were carried out twice per day from 1–4 dpi. After infection, pH values and glucose concentrations were only measured before harvest and medium exchange. Under these conditions, smaller volumes of NaHCO_3_ and glucose were required to maintain pH values and glucose concentrations at similar levels as in experiment 4 (Figure 2A, Table 1); however, pH values and glucose concentrations remained difficult to control at 1 and 2 dpi.

A peak infectivity titer of 6.4 log_10_ TCID_50_/mL was observed for the harvest at 52 hpi for experiment 5 (Figure 2D). Three harvests had infectivity titers ≥6 log_10_ TCID_50_/mL (harvests at 38, 52 and 62 hpi). The accumulated TCID_50_ from all harvests (3.9 L) was 9.5 log_10_ TCID_50_, resulting in a CSVY of 1.3 TCID_50_/cell (Table 3). The virus yield from this culture with two harvests per day was thus approximately 1.6-fold increased, compared to the yield from the first culture with one harvest per day (experiment 4).

In a similar experiment with two harvests and medium exchanges per day at 1–3 dpi (experiment 6), a peak infectivity titer of 7.0 log_10_ TCID_50_/mL was observed for the harvest at 60 hpi (Figure 2D). Three harvests had infectivity titers ≥6.5 log_10_ TCID_50_/mL (harvests at 36, 49, and 60 hpi), and two additional harvests had infectivity titers in the range 6–6.5 log_10_ TCID_50_/mL (harvests at 24 and 73 hpi). The accumulated TCID_50_ from all harvests was 10.1 log_10_ TCID_50_ (3 L), resulting in a CSVY of 5.9 TCID_50_/cell (Table 3). The virus yield from this second culture with two harvests per day (experiment 6) was approximately 4.3-fold higher than that of the first culture with two harvests per day (experiment 5) and 7.1-fold higher when compared to the yield of the culture with one harvest per day (experiment 4). Deep sequencing of virus from experiment 5 harvested at 38 hpi showed viral genetic stability. Compared to the seed virus there were only two substitutions in the Spike protein, R685S and S686R, both recorded with frequencies of 5–10% (Table 2). Overall, two harvests per day allowed better control of glucose concentration and pH values and increased virus yields.

### 3.4. Virus Production at 33 °C Further Enhanced Virus Yield (Experiment 7)

**Figure 3:**
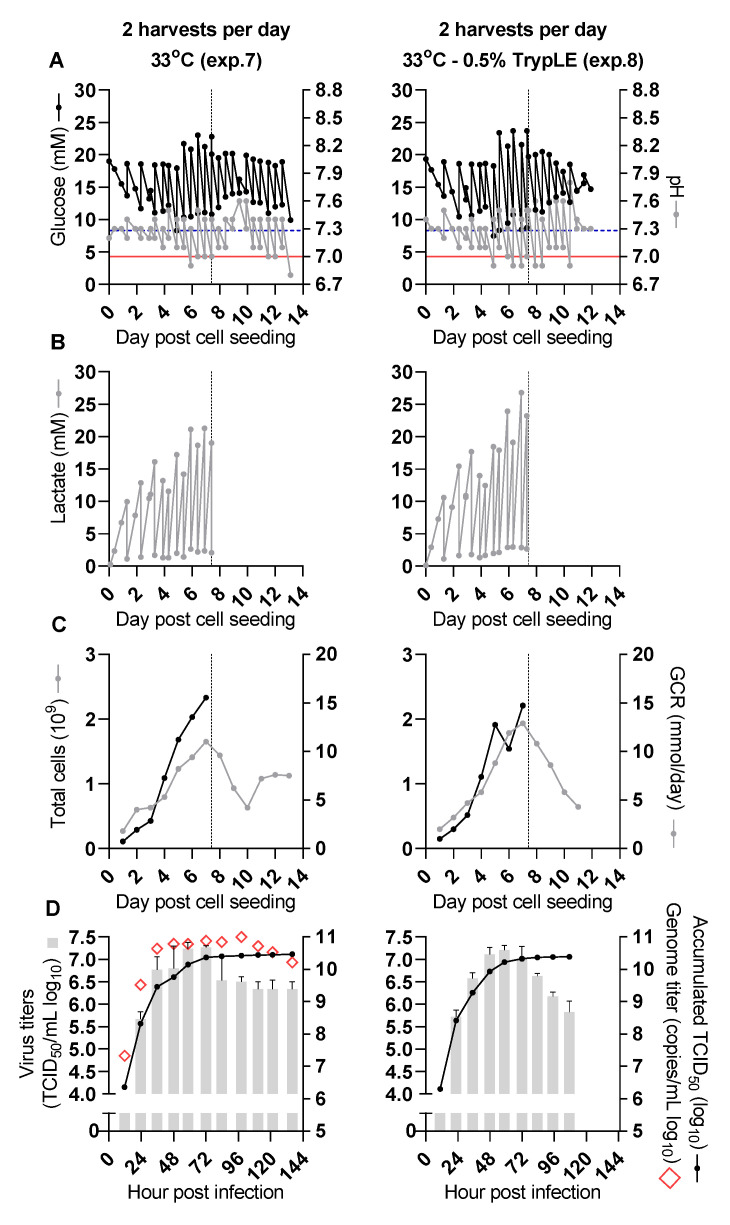
SARS-CoV-2 production was improved by a temperature reduction to 33 °C but not by the addition of 0.5% TrypLE (experiment 7 and 8). Before the time of infection (dashed vertical line), pH values and concentrations of glucose and lactate were measured before and after adjustment at each time point. After infection, glucose concentrations and pH values were measured before and after each harvest. Harvests were carried out twice per day, and cultures were maintained without (experiment 7, left panels) or with (experiment 8, right panels) 0.5% (*v/v*) TrypLE added to the cell culture medium. The two experiments were done independently. (**A**) Cell culture supernatant glucose concentrations (black dots) and pH values (grey dots); the dashed blue horizontal line represents a glucose concentration of 8.3 mM, the red horizontal line represents a pH value of 7, indicating the desired lower limits for these parameters. (**B**) Cell culture supernatant lactate concentrations. (**C**) Total cell numbers per CelCradle^TM^ 500-AP calculated from a sample of five carriers (black dots) and GCR (grey dots). (**D**) RNA titers of individual harvests (red diamonds, only evaluated for experiment 7), infectivity titers of individual harvests (grey columns, showing the geometric mean of triplicate titrations and error bars indicating standard error of the mean), and SARS-CoV-2 yield expressed as accumulated TCID_50_ units (black dots, sum of TCID_50_ units obtained from each harvest of an experiment).

It has been described for both protein and virus production that a reduction in temperature may have beneficial effects on product yields [25,26,27]. Therefore, after cell seeding, expansion, and infection as described above (Figure 3A–C), a culture was maintained at 33 °C with virus harvest as well as medium exchange being carried out twice per day at 1–6 dpi. In this and the following experiment pH values and glucose concentrations were measured both before and after harvest and medium exchange. Compared to the previous experiments, following infection, the pH values and glucose concentrations were better maintained above the lower target levels with a cultivation temperature of 33 °C (Figure 3A, Table 1).

A peak infectivity titer of 7.3 log_10_ TCID_50_/mL was observed for the harvest at 72 hpi (Figure 3D). Thus, six harvests had infectivity titers ≥6.5 log_10_ TCID_50_/mL (harvests at 36, 48, 59, 72, 84 and 98 hpi), and three additional harvests had infectivity titers in the range 6–6.5 log_10_ TCID_50_/mL (harvests at 111, 122 and 136 hpi). The accumulated TCID_50_ from all harvests (5.2 L) was 10.5 log_10_ TCID_50_, resulting in a CSVY of 12.7 TCID_50_/cell (Table 3). The total virus yield was thus increased approximately 9.4- and 2.2-fold, when compared to the yields obtained with two harvests per day at 37 °C in experiments 5 and 6, respectively. The peak RNA titer of 11 log_10_ copies/mL was observed for the harvest at 98 hpi (Figure 3D). Harvests at 36 to 111 hpi had RNA titers in the range of 10.7–11 log_10_ copies/mL, and a total of 2.4 × 10^14^ copies were obtained from this experiment. Approximately 3.7 mg Spike protein subunit S1 in total was obtained from a pool of eight harvests with the highest infectivity titers from 36 to 122 hpi according to S1 quantification in chemically inactivated samples by ELISA as described in Materials and Methods. Deep sequencing of an inactivated virus pool (experiment 7 virus pool) consisting of these eight harvests from experiment 7 collected at 2–5 dpi and spiked with virus from experiment 5 harvested at 62 hpi indicated one substitution in the Spike protein, S686R, compared to the seed virus with a frequency of 5% (Table 2).

### 3.5. Addition of TrypLE to the Cell Culture Medium Did Not Enhance Virus Production (Experiment 8)

It has been suggested that trypsin activity is important for SARS-CoV-2 entry and that exogenously added trypsin may enhance SARS-CoV-2 spreading in cell culture [28,29,30]. To confirm these results using our virus and cell line, we tested in T25 flasks whether the addition of TrypLE, a recombinant trypsin replacement product, to the cell culture medium had an effect on virus yields at 37 °C. TrypLE concentrations in the range of 0.25–1% (*v/v*) were evaluated. A concentration of 0.5% (*v/v*) TrypLE improved virus spreading and resulted in slightly higher infectivity titers, whereas a concentration of 1% (*v/v*) TrypLE led to significant cell death and reduced infectivity titers compared to the culture without TrypLE. A concentration of 0.25% (*v/v*) TrypLE resulted in similar cell death and infectivity titers as observed in the culture without TrypLE (data not shown). Also, in non-infected cells maintained in T25 flasks at 33 °C, 1% (*v/v*) TrypLE resulted in moderate cell death whereas 0.5% (*v/v*) TrypLE did not result in apparent cell death. Trying to further improve the culture conditions so far resulting in the highest virus yields (experiment 7), we evaluated if 0.5% (*v/v*) TrypLE also enhanced infectivity titers and overall virus yield in a CelCradle^TM^ 500-AP culture maintained at 33 °C after the time of infection. For this, cells were expanded in the CelCradle^TM^ 500-AP as described above and infected at 7 dpcs. From the time of infection, the culture was maintained at 33 °C and 0.5% (*v/v*) TrypLE was added to the culture medium upon each harvest and medium exchange. Harvest and medium exchange were carried out twice per day at 1–5 dpi. Additional NaHCO_3_ would have been required to keep pH above the lower target level at 1 dpi, however, pH values and glucose concentrations were generally well maintained as was also the case during the previous 33 °C virus production (experiment 7) (Figure 3A, Table 1). 

A peak infectivity titer of 7.2 log_10_ TCID_50_/mL was observed for the harvest at 59 hpi (Figure 3D). Five harvests had infectivity titers ≥6.5 log_10_ TCID_50_/mL, (harvests at 35, 48, 59, 72 and 83 hpi), and one additional harvest had infectivity titers in the range 6–6.5 log_10_ TCID_50_/mL (harvest at 95 hpi). The accumulated TCID_50_ from all harvests (4.3 L) was 10.4 log_10_ TCID_50_, resulting in a CSVY of 11.1 TCID_50_/cell (Table 3). Overall, the virus yield from this culture maintained at 33 °C with 0.5% TrypLE in the cell culture medium during the virus production phase was not improved compared to the culture without TrypLE (experiment 7), which had approximately 1.2-fold higher yield.

## 4. Discussion

Efficient virus production is critical for vaccines based on whole viruses but also has applications in basic virus research, such as for ultrastructural studies of viral particles, animal immunogenicity studies, vaccine challenge trials, and in development of diagnostic assays. In this study, we established efficient SARS-CoV-2 production in animal component-free conditions using a well-characterized cell line cultivated in the CelCradle^TM^ 500-AP bioreactor, a scalable cell culture system useful for process development and optimization studies for production of vaccines. Further, the established procedures could be of value for generation of virus seed stocks for subsequent pilot and production scale vaccine manufacturing.

Traditionally, adherent cells have been cultivated in monolayers applying roller bottles or multilayer cell culture flasks for production purposes. For adherent cells that are difficult to cultivate on microcarriers in stirred tank bioreactors, options for large-scale high-density cell culture have been limited. Packed-bed bioreactors facilitating large-scale cultivation of adherent cells have recently been developed. The CelCradle^TM^ 500-AP (Esco Aster Pte. Ltd.) is a packed-bed bioreactor with a 0.5 L working volume operated by tide-motion for mixing with a bed consisting of BioNOC™ II macrocarriers. It thus supports cultivation of adherent cells and operates with a gentle mixing mode. The CelCradle^TM^ 500-AP has been demonstrated to successfully support production of Japanese encephalitis virus in Vero cells [31], hepatitis D virus-like particles in baby hamster kidney cells [32], and influenza A virus in Madin-Darby canine kidney cells and Vero cells [33]. Importantly, the CelCradle^TM^ 500-AP used in this study is a laboratory-scale version of the TideXcell system available with packed-bed capacities of up to 100 L, thus applying the cultivation principles of the CelCradle^TM^ 500-AP at production scale [34]. A linear scalability from the CelCradle^TM^ to the 10 L TideXcell system was recently reported for influenza A virus in Madin-Darby canine kidney cells [33]. Apart from the TideXcell systems, other large-scale packed-bed bioreactors available include the iCELLis 500+ system (Pall Life Sciences, New York, NY, USA) and scale-X nitro (Univercells Technologies, Nivelles, Belgium), both with culture volumes of 60–70 L [14].

According to recent reports, Vero (WHO) cell culture-derived SARS-CoV-2 for the inactivated whole virus vaccines from Sinovac [24] and Sinopharm [35] were produced in a 25 L wave bag bioreactor (Cytiva) [36] and a 10 L basket bioreactor [35,37], respectively. The inactivated whole virus vaccine from Bharat Biotech was produced in unspecified bioreactors [38,39], while the vaccine developed by Valneva currently going through clinical trials is produced in roller bottles [40]. Basket bioreactor cultures were reported to yield harvest titers of around 7 log_10_ TCID_50_/mL, but no process details have been published describing virus production in these bioreactors. The wave bag bioreactor technology might be scaled to working volumes of up to 100 L, and while the basket bioreactor system is now used in large-scale vaccine manufacturing, specific information on reactor size and working volume is not publicly available. More detailed reports of cell-culture based SARS-CoV-2 production could guide improvements of virus production processes.

SARS-CoV-2 production in this study was based on a highly characterized Vero (WHO) cell bank also available for GMP production [41]. It is derived from the WHO Vero cell bank which is widely accepted for vaccine production and used in several licensed viral vaccines based on cell-derived antigen [42], including the inactivated SARS-CoV-2 vaccines mentioned above. These cells show high permissiveness to SARS-CoV-2 infection and can readily be grown in serum-free medium. The total cell numbers of Vero cells in the CelCradle^TM^ 500-AP cultures obtained with the culture conditions of this study were similar to those in previous reports on Vero cell expansion in the CelCradle^TM^ 500-AP bioreactor [31,43]. Different harvest strategies and production conditions were tested in this study, and the highest yield was obtained with two harvests per day and a change of temperature to 33 °C at the time of infection (experiment 7). While production at 33 °C was reported to increase yield in other bioprocesses, it is possible that the improved virus yield observed at 33 °C in this study was at least partly attributed to improvements in pH control due to changes in cell metabolism at the reduced temperature [25,27,44]. In T25 flask experiments in our laboratory, typically peak infectivity titers of up to 6.5 log_10_ TCID_50_/mL and CSVY of up to 7 have been observed. Thus, virus production at improved conditions in the CelCradle^TM^ 500-AP appeared more efficient than production in small monolayer cell cultures.

Limited consumption of glutamine was observed, and thus glucose was the main carbon source of the culture, which was also reflected in the low levels of ammonia and relatively high levels of lactate measured in the culture supernatant. Lactate levels in the range of 20–30 mM, measured in this study, have been reported not to considerably interfere with cell growth in other studies [45,46]. However, cell culture parameters with undetectable effects on cell growth could potentially still affect cell culture productivity [44]. Additionally, during cell expansion, it became challenging to maintain pH within the desired range by manual addition of NaHCO_3_. Thus, further improved productivity might be achieved in a bioreactor supporting closed-loop pH control to compensate for the acidification by the lactate. Recently developed improved versions of the CelCradle^TM^ 500-AP and the larger scale TideXcell systems are equipped with closed-loop pH control and thus enable a stable pH during the period of cell culture and virus propagation.

Whereas trypsin-like protease activity plays a role for SARS-CoV-2 cell entry [28,29,30], addition of 0.5% (*v/v*) TrypLE to the culture medium (experiment 8) from the time of infection did not increase virus production in the CelCradle^TM^ 500-AP culture maintained at 33 °C. It should be mentioned that TrypLE activity is sensitive to pH and temperature [47]. The low pH values measured at 1 dpi, possibly in combination with the cultivation temperature of 33 °C, might thus reduce TrypLE activity and potentially reduce the effect on virus yield when compared to the pilot experiment in T25 flasks at 37 °C. In future experiments, it would be relevant to test the effect of TrypLE addition on virus production in a CelCradle^TM^ 500-AP culture maintained at 37 °C.

In addition to application of closed-loop pH control, other strategies to further improve the virus yield from Vero cells in this packed-bed bioreactor could target cell culture medium formulation or additives [25,48]. Further, optimized medium refreshment strategies during cultivation to reduce waste accumulation and maintain nutrient levels, such as optimized schemes for introduction of partial medium exchange, cell culture medium recirculation or perfusion, might contribute to increased productivity [33,46,49,50,51,52]. These feeding modes could similarly benefit pH control. A further cell culture adapted SARS-CoV-2 virus inoculum might also facilitate higher production yields. It is possible that other SARS-CoV-2 strains show different growth characteristics in Vero cells, thus, producing other strains of SARS-CoV-2 in the CelCradle^TM^ 500-AP could require adjustments of the protocols described in this study to optimize yield.

The SARS-CoV-2 virus strain used in this study was isolated from a Danish patient [15] and had six amino acid changes compared to the Wuhan-Hu-1 SARS-CoV-2 virus strain [53] (Table 2). The Danish isolate carries the D614G substitution, presenting the po-lymorphism that has been dominating at this position globally since the spring of 2020 [54]. Of interest for vaccine antigen production, the D614G substitution has been reported to increase virion Spike protein content and to mediate increased infectivity [55]. The four substitutions in the S protein in the CelCradle^TM^ 500-AP virus seed stock with at least 5% prevalence acquired during four passages in cell culture were represented in the GISAID database of patient-derived virus sequences [56], however, at frequencies <0.2% (total number of registered sequences were 415,516, as of 12 April 2021). These substitutions were not located in the critical receptor binding domain of the Spike protein. It will be relevant to investigate immunogenicity of the CelCradle^TM^ 500-AP produced SARS-CoV-2 and to evaluate how substitutions in the vaccine antigen Spike protein might influence immunogenicity and protection against COVID-19 conferred by relevant virus variants [57].

Taken together, our data show that with a small footprint laboratory-scale bioreactor SARS-CoV-2 can be produced at high titers of up to 7.3 log_10_ TCID_50_/mL, with an accumulated TCID_50_ of 10.5 log_10_ TCID_50_, and obtaining 3.7 mg S1 from one experiment. Assuming a vaccine dose of 7 log_10_ TCID_50_ or 1 ug S1 and 50% loss in downstream processing, approximately 1500 doses could be obtained from one CelCradle^TM^ 500-AP culture (1 dose / 3 mL bioreactor harvest) and several hundred thousand vaccine doses could potentially be obtained from a 100 L TideXcell bioreactor. Assumptions on the vaccine dose are based on data for other antiviral vaccines [36,37,58,59,60,61,62] and observations from preliminary immunogenicity studies in mice (unpublished). Virus manufacturing based on the described and similar processes might be of interest for inactivated but also attenuated second-generation COVID-19 vaccines [63,64].

## Figures and Tables

**Table 1:** CelCradle^TM^ 500-AP experiments, cell culture parameters.

Experiment	1	2	3	4	5	6	7	8
Harvests per day	na	na	na	1	2	2	2	2
Temperature post infection, °C	na	na	na	37	37	37	33	33
Additives post infection	na	na	na	none	none	none	none	TrypLE
**Cell numbers** **prior to infection**								
Seed cell number	1.4 × 10^8^	2.3 × 10^8^	1.5 × 10^8^	1.5 × 10^8^	1.5 × 10^8^	1.5 × 10^8^	1.5 × 10^8^	1.5 × 10^8^
Peak total cell number	2.8 × 10^9^	2.7 × 10^9^	2.7 × 10^9^	2.5 × 10^9^	2.3 × 10^9^	2.3 × 10^9^	2.3 × 10^9^	2.2 × 10^9^
Cells/carrier	3.5 × 10^6^	3.4 × 10^6^	3.4 × 10^6^	3.1 × 10^6^	2.9 × 10^6^	2.8 × 10^6^	2.9 × 10^6^	2.7 × 10^6^
Cells/mL	5.6 × 10^6^	5.4 × 10^6^	5.4 × 10^6^	5.0 × 10^6^	4.7 × 10^6^	4.6 × 10^6^	4.7 × 10^6^	4.4 × 10^6^
Peak total cell number, dpcs	9	9	9	7 ^a^	7 ^a^	7 ^a^	7 ^a^	7 ^a^
**Cell culture supernatant parameters** **prior to infection**								
Peak GCR, mmol/day (dpcs)	11 (9)	12 (8)	13 (7)	13 (7)	13 (7)	12 (7)	11 (7)	13 (7)
Glucose range, mM	7.7–22	7.1–23	9.5–25	8.7–29	7.8–26	8.4–24	8.3–23	7.5–24
Lactate, mM ^b^	24	26	25	27	25	25	21	27
Glutamine range, mM ^c^	3.5–4.2	3.7–4.1	3.7–4.1	nd	nd	nd	nd	nd
Ammonia range, mM ^c^	0.1–0.4	0.1–0.5	0.1–0.6	nd	nd	nd	nd	nd
pH range	6.9–7.5	6.8–7.5	6.9–7.5	6.9–7.5	7–7.5	7–7.5	6.9–7.5	6.9–7.5
**Cell culture supernatant parameters** **post infection**								
Glucose range, mM	na	na	na	6.7–27	4.9–25	8.1–20	9.9–20	11–21
pH range	na	na	na	6.8 ^d^	6.8 ^d^	6.7–7.8	6.8–7.6	6.9–7.8

dpcs: days post cell seeding, GCR: glucose consumption rate, na: not applicable, nd: not determined. ^a^ Cultures were infected at 7 dpcs, cell numbers were not determined post-infection. ^b^ The highest lactate concentration measured during cultivation. ^c^ Evaluated in selected experiments. ^d^ pH values were only measured before medium exchange/harvest.

**Table 2:** Deep sequencing of the CelCradle^TM^ 500-AP virus seed stock and selected harvests.

Nucleotide Position ^a,b^	Reference Nucleotide ^a^	Alternative Nucleotide	Prevalence of Nucleotide Change			Gene	Amino Acid Change ^d^
			Seed Stock	Exp. 5 ^c^	Exp. 7 Virus Pool ^c^		
**1059**	**C**	**T**	**100**	**100**	**100**	**nsp2**	**T85I**
**14,408**	**C**	**T**	**99**	**100**	**100**	**nsp12**	**P323L**
21,759	A	G	47	45	40	S	H66R
22,206	A	G	12	11	8	S	D215G
22,296	A	G	17	11	15	S	H245R
22,301	A	C	5	9	10	S	S247R
**22,487**	**G**	**A**	**14**	**16**	**17**	**S**	**E309K**
**23,403**	**A**	**G**	**100**	**100**	**100**	**S**	**D614G**
23,615	C	A	2	5	4	S	R685S
23,618	A	C	3	7	5	S	S686R
**25,563**	**G**	**T**	**100**	**100**	**100**	**ORF3a**	**Q57H**
**28,899**	**G**	**T**	**99**	**99**	**100**	**N**	**R209I**

Exp.: Experiment. ^a^ Nucleotide position relating to the reference SARS-CoV-2 genome sequence: GenBank accession number NC_045512. Coding nucleotide changes occurring in at least one of the analyzed virus populations with ≥5% prevalence are listed. Spike protein-encoding region of NC_045512: nucleotide position 21,563–25,384. ^b^ Bold font indicates mutations present at consensus level in the virus isolated from the patient. ^c^ Exp. 5: Exp. 5, harvest at 38 hpi. Exp. 7 virus pool: pooled harvests from Exp. 7 at 36–122 hpi and Exp. 5 at 62 hpi. ^d^ The position is the amino acid number in the indicated gene.

**Table 3:** CelCradle^TM^ 500-AP experiments, virus yield.

Experiment	4	5	6	7	8
Harvests per day	1	2	2	2	2
Temperature post infection, °C	37	37	37	33	33
Additives post infection	none	none	none	none	TrypLE
Total harvest volume, L ^a^	2	3.9	3	5.2	4.3
Peak infectivity titer, log_10_ TCID_50_/mL (hpi)	6.5 (46)	6.4 (52)	7.0 (60)	7.3 (72)	7.2 (59)
Total accumulated TCID_50_ units, log_10_ ^b^	9.3	9.5	10.1	10.5	10.4
CSVY, TCID_50_ units/cell	0.8	1.3	5.9	12.7	11.1
Yield compared to experiment 7 ^c^	7%	11%	46%	100%	83%

hpi: hours post-infection, CSVY: cell-specific virus yield. ^a^ Individual harvest volumes of 0.45–0.5 L. ^b^ TCID_50_ units obtained from each harvest were calculated from the average harvest infectivity titer and the harvest volume. The total accumulated TCID_50_ units from each experiment are given as the sum of TCID_50_ units obtained in each harvest of the experiment. ^c^ Calculated from the total accumulated TCID_50_ units.

## Data Availability

Not applicable.
